# Spinal Cord Stimulator Therapy as a Last Resort Option in Refractory Neuropathic Groin Pain: A Case Report

**DOI:** 10.7759/cureus.33098

**Published:** 2022-12-29

**Authors:** Gurtej Bajaj, Gaurav Gupta, Warren A Southerland, Daniel Southren

**Affiliations:** 1 Physical Medicine and Rehabilitation, University of Pennsylvania, Philadelphia, USA; 2 Anesthesiology and Critical Care/Pain Medicine, Hospital of the University of Pennsylvania, Philadelphia, USA; 3 Pain Medicine, Minivasive Pain & Orthopedics, Houston, USA; 4 Anesthesiology, Mount Auburn Hospital, Harvard Medical School, Boston, USA

**Keywords:** refractory pain, post hernia repair, case report, groin pain, spinal cord stimulator

## Abstract

A spinal cord stimulator (SCS) is an excellent therapeutic strategy for many refractory chronic pain cases. They have a multi-faceted effect in providing relief in such indicated cases that include complex regional pain syndrome (CRPS) or failed back surgery syndrome (FBSS). However, if a patient has chronic intractable neuropathic pain outside the standard indications for SCS, can a case be made for trialing SCS as a last resort option? We describe a case where a patient with neuropathic groin pain, refractory to numerous types of procedures and non-interventional modalities, successfully underwent SCS therapy as a last resort option.

## Introduction

A spinal cord stimulator (SCS) is an excellent therapeutic strategy in many refractory chronic pain cases, including complex regional pain syndrome (CRPS) and failed back surgery syndrome (FBSS) [1]. Fewer than half of all patients with neuropathic pain are adequately controlled with pharmacotherapy alone [2]. In this setting, minimally invasive procedures have become increasingly utilized, and spinal cord stimulators have been no exception.

The FDA-approved indications for the use of SCS include CRPS, FBSS, and intractable lower back, leg, trunk, or limb pain [3]. Though this covers a wide variety of symptoms and conditions, they tend to be more clearly defined before implantation. However, if a clear etiology cannot be elicited for a patient who has neuropathic groin pain, the role of SCS has yet to be described. In this case, we discuss a patient with intractable groin pain of unclear etiology who achieved long-term relief with SCS therapy.

## Case presentation

A 69-year-old male with a past medical history of hypertension, irritable bowel syndrome, and generalized anxiety disorder presented with refractory left groin pain status post bilateral laparoscopic inguinal hernia repair. He began to have left groin pain that radiated into his testicles after his surgery. He described it as a “low-grade ache” at rest and increased in intensity during exercise and stretching. The patient lives an active lifestyle, so the pain was interrupting his daily life. An MRI pelvis did not show a recurrence of the inguinal hernias or any other significant findings.

Prior to the presentation at our clinic, he was evaluated by another pain specialist, who started him on physical therapy and over-the-counter medications, including acetaminophen and ibuprofen. Physical therapy worsened his pain. Acetaminophen, ibuprofen, and ice helped the pain minimally. He underwent two left genitofemoral nerve injections with excellent short-term relief. Subsequently, a pulsed radiofrequency ablation was performed on the left genitofemoral nerve, which caused him increased pain.

Upon his initial visit, the exam revealed tenderness over the left groin scar. The patient was started on duloxetine for neuropathic pain and was prescribed a transcutaneous electric nerve stimulation (TENS) unit. The patient failed duloxetine due to gastrointestinal side effects. At his next visit, he stated that he did not want to pursue medical management, so an ilioinguinal and iliohypogastric nerve block was recommended and scheduled. The patient underwent two ilioinguinal and iliohypogastric nerve blocks over two months, both of which were performed under ultrasound guidance and used 9 mL of 0.25% bupivacaine and 10 mg of dexamethasone. The first nerve block provided 100% pain relief for 12 days and benefited the patient for two months. The second nerve block was effective but provided less pain relief for a shorter amount of time.

**Figure 1:**
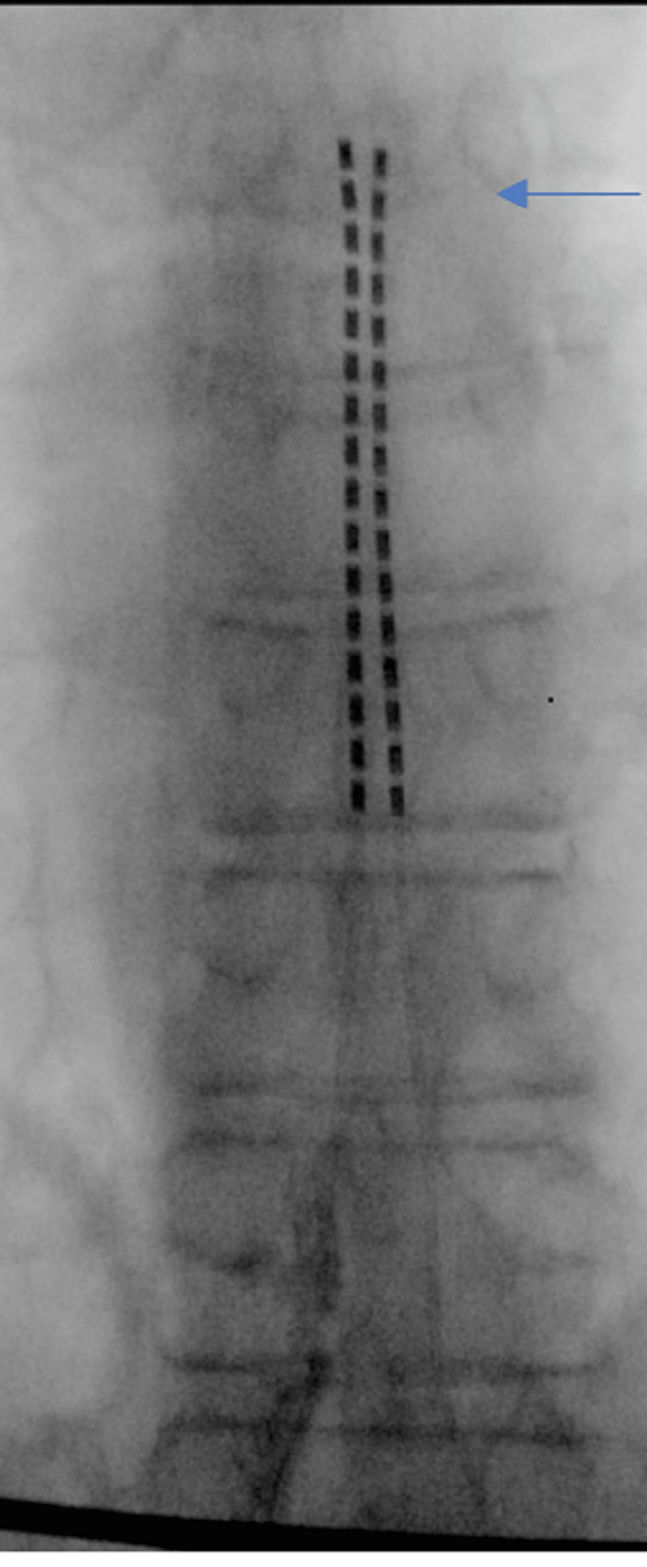
The arrow points to the superior end plate of T7 of the bilateral SCS trail leads that alleviated the patient's groin pain SCS: Spinal cord stimulator

Given the refractory response to the numerous therapies, both interventional and pharmacological, the patient elected to pursue spinal cord stimulation. A trail SCS with the stimulator leads was placed at the T7 superior endplate bilaterally (Figure 1) and was deemed successful when at his post-operative follow-up he reported that he had no pain with exercising, stretching, and increasing mobility. A permanent Boston Scientific SCS was implanted and there were no surgical complications. 

During his post-implantation follow-up visits, the patient endorsed no pain at rest, minimal discomfort while lying supine (due to the battery), and great pain relief with the focused assessment with sonography for trauma (FAST) programming while exercising. He did report one episode of pain during exercise, which was corrected by reprogramming. A thoracic spine radiograph showed that both leads traveled superiorly to the T4-5 range. The patient on subsequent follow-up visits reported excellent ongoing, complete pain relief with increased mobility and quality of life. He typically turns on the SCS only when he works out, which continues to provide 100% pain relief.

## Discussion

Chronic inguinal pain after inguinal note repair has been observed in 14% to 54% of patients, with 1% to 2% of these patients noting refractory pain that affects their quality of life [4]. The incidence of chronic inguinal pain has been quantified to be 8,000 to 16,000 cases per year [5].

Neuropathic pain from inguinal hernia repair is the most common pain type appreciated in the literature (30% to 50% of all cases), but somatic and visceral have also been recorded [4,6]. Damage to the nerves by ligation or crush injury during repair is the etiology of the burning pain that can radiate into the inner thigh and to the scrotum. Treatment of chronic groin pain is difficult. Some options include oral analgesics, nerve blocks (ilioinguinal, iliohypogastric, and genitofemoral nerves) neurolysis, peripheral nerve stimulation, and spinal cord stimulation [7]. Differentiating the exact nerve causing the symptoms can be difficult to ascertain given the overlap of the genitofemoral and ilioinguinal nerves. Selective blocks temporarily spaced can help differentiate the causative nerve, but it is only if surgical resection is to be pursued [8]. Navigating the next course of action if conservative means fail, however, has not been appropriately delineated at this time.

Currently, there is no strong evidence regarding a particular threshold of failed therapies before pursuing SCS. An SCS is known to be an effective intervention in providing relief for refractory neuropathic groin pain and delaying an appropriate intervention for such patients will negatively impact their quality of life. In this case, alleviation of pain by non-SCS modalities was temporary or made pain symptoms worse, but upon SCS implantation, there was 100% pain relief that allowed the patient to continue with his passion for exercising.

Few case reports have shown a significant reduction in groin pain from hernia repair after SCS placement. Elias demonstrated two patients who endured at least one year of neuropathic groin pain while failing oral, non-opioid medications, and regional nerve blocks [9]. Of note, the pain was bothersome enough that opioid medications were initiated. Following SCS implementation, there was a greater than 50% reduction in both patients’ pain levels leading to complete cessation of scheduled opioid administration in one patient and a 40% reduction in methadone dose without the need for breakthrough analgesia in the other patient [9]. Though SCS is proven to alleviate refractory pain conditions, there is a great variation as to the determination of other options and treatments that have failed among physicians and patients alike [10]. In this case, the patient had failed multiple procedures and pharmacotherapy prior to concluding that SCS should be trialed.

There are possible explanations as to why SCS may have worked more definitively compared to the previous procedures. First, SCS is an ongoing, consistent therapy as opposed to a one-time procedure, even if it involves attempts at definitive therapy (i.e., ablation). Second, it acts more centrally compared to other therapies that the patient underwent. Not only can SCS act at the spinal level, but it has also been shown to exert effects at the cortical level, both in instances of neuropathic pain and spasticity [11]. One report by Sufianov et al. in 2014 showed via electroencephalogram (EEG) and positron emission tomography (PET) studies the reversal of pathologic metabolic activity in the cortex post-SCS implantation [12]. This multi-level coverage certainly gives SCS an advantage over other therapy modalities.

Ultimately, more studies are needed to better understand when SCS therapy should be pursued within the therapeutic process. Though every effort should be made to determine the etiology of a patient's pain and less invasive steps should be pursued initially, SCS therapy should be considered in patients with refractory groin pain.

## Conclusions

Although SCS has been an effective tool in the chronic pain therapeutic paradigm, there is no strong data regarding a particular threshold of failed therapies before pursuing SCS currently. Furthermore, for conditions that may not necessarily fall under the umbrella of indications, an SCS can be considered or discussed as a last resort option given its multitude of targets at both the spinal and cortical levels. As illustrated in this case, the SCS was the only treatment that provided the patient with substantial analgesia to enable him to return to his exercise routine. Ultimately, high-quality studies are needed to better delineate when to initiate SCS therapy for nontraditional pain indications and analyze its efficacy for such cases.
